# The efficacy and safety of rituximab monotherapy in the new onset pediatric idiopathic nephrotic syndrome: a randomized controlled clinical trial

**DOI:** 10.1080/0886022X.2025.2499902

**Published:** 2025-05-06

**Authors:** Ai-Qin Sheng, Fei Liu, Qiu-Yu Li, Ya-Lan Dou, Xiao-Jing Zhang, Jing-Li Zhao, Ling-Fei Huang, Si-Yi He, Zhi-Hong Lu, Chun-Yue Feng, Jing-Jing Wang, Hui-Jun Shen, Hai-Dong Fu, Wei-Li Yan, Jian-Hua Mao

**Affiliations:** ^a^ Department of Nephrology, Children’s Hospital, Zhejiang University School of Medicine, National Clinical Research Center for Child Health, Hangzhou, China; ^b^ Department of Clinical Epidemiology & Clinical Trial Unit, Children’s Hospital of Fudan University, National Children’s Medical Center, Shanghai, China; ^c^ Department of Pharmacy, Children’s Hospital, Zhejiang University School of Medicine, National Clinical Research Center for Child Health, Hangzhou, China

**Keywords:** Nephrotic syndrome, children, rituximab, CD20 monoclonal antibody, glucocorticoids, outcome

## Abstract

**Background:**

Nephrotic syndrome (NS) is a common form of glomerular disease in children, characterized by a high propensity for relapse. Prolonged use of glucocorticoids (GCs) can result in various side effects. Rituximab (RTX) may be considered as an initial treatment option for primary nephrotic syndrome in pediatric patients.

**Methods:**

We conducted a prospective, single-center, randomized controlled trial (RCT) to evaluate whether the initial use of RTX monotherapy is superior to GC therapy in treating pediatric idiopathic NS and to assess its safety. The primary outcome and secondary outcomes were compared between the two groups.

**Results:**

A total of 24 pediatric patients were included in the study, comprising 19 males and 5 females. After six weeks of treatment, the complete remission (CR) rate in the GC group was significantly higher than that in the RTX group (100% vs 33.3%). Compared with the RTX group, the GC group had a shorter time to first remission (14.25 d vs. 9.5 d). During the follow-up period, none of the patients in the RTX group who achieved CR experienced relapse, with the longest relapse-free duration being 79 weeks. In the GC group, nine patients experienced relapse with the longest relapse-free period being 94 weeks. No serious adverse events occurred in either group. The cumulative steroid dosage was not statistically different between the 2 groups (*p* = 0.41).

**Conclusion:**

Although the CR rate of children with idiopathic NS treated with RTX alone is significantly lower, the relapse rate among responders to RTX is also lower than that of the GC treatment.

## 1. Introduction

Nephrotic syndrome (NS) is the most prevalent glomerular disease in children, with idiopathic nephrotic syndrome (INS) comprising over 90% of cases. The incidence of INS ranges from approximately 1.15 to 16.9 per 100,000, varying by ethnicity and region [1]. Notably, the incidence in Asia is higher than that in Europe and North America [2]. A meta-analysis indicates that the annual average incidence of INS is 2.92 per 100,000 [3]. Currently, corticosteroid therapy is the first-line treatment for pediatric INS, with approximately 85% of patients achieving complete remission (CR) of proteinuria within 4–6 weeks of adequate steroid use, which is defined as steroid-sensitive NS (SSNS). However, around 70–80% of steroid-sensitive patients experience disease relapse during steroid tapering, with about 50% showing frequent relapses or steroid dependency [1,3]. This situation requires repeated use of steroids in conjunction with other immunosuppressive agents, including tacrolimus, cyclosporine, cyclophosphamide, and mycophenolate mofetil. Long-term, repeated, and high-dose steroid use may result in obesity, growth retardation, hirsutism, skin striae, and so on. In this case, it affects the psychological well-being of patients and their families. Additionally, it increases the risk of gastrointestinal ulcers, hypertension, glaucoma, osteoporosis, infections, and diabetes [4]. Therefore, the long-term objective of NS treatment is to avoid relapses, minimize drug side effects, and enhance the quality of life.

Disruption of immune cells and circulating immune factors leads to abnormalities and damage in podocyte structure and function, impairing the glomerular filtration barrier. This is a significant pathogenic mechanism of INS, although the exact mechanisms remain unclear [2]. It was previously believed that T cell dysfunction plays a critical role. Chemokines and cytokines released by T cells upon stimulation, such as TNF-α, IL-4, and IL-13, can increase glomerular filtration membrane permeability, induce inflammatory responses, and subsequently result in proteinuria [5]. The number and function of regulatory T cells (Treg cells) may decline, leading to immune imbalance and an increased immune response that damages the glomerular filtration membrane [6]. In recent years, it has become increasingly recognized that abnormal B cells function may play a more prominent role. Research has demonstrated a strong correlation between the quantity of B cells and the state of the INS disease, with B cell counts rising during relapses and falling during remissions [7].

Rituximab (RTX) is a chimeric CD20 monoclonal antibody that specifically binds to CD20 molecules on the surface of pre-B and mature B cells, eliminating B cells through antibody-dependent cytotoxicity, complement-dependent pathways, and direct induction of B cell apoptosis. This mechanism reduces antibody production in circulation and inhibits the interaction between B and T cells, leading to disease remission. While RTX induces B cell depletion, it can also increase the number of Treg cells [8]. Furthermore, RTX can bind to sphingomyelin phosphodiesterase acid-like 3b (SMPDL-3b) on podocyte surfaces, regulating acidic sphingomyelinase (ASMase) activity and preventing actin cytoskeletal degradation and podocyte apoptosis [9,10]. Moreover, some studies have indicated that RTX is involved in cytokine regulation, although the specific mechanisms remain unclear. Over the past decade, RTX has been increasingly utilized in the management of INS. Multiple studies have demonstrated that RTX is well tolerated in the treatment of pediatric NS. This treatment does not require long-term oral medications, and effectively reduces steroid dependence or frequent relapses. Furthermore, it reduces the use of steroids in patients with steroid-resistant nephrotic syndrome, resulting in lower relapse rates and extended relapse-free survival [11,12].

Fenoglio et al. and Xu et al. [13,14] have validated the efficacy and safety of RTX in treating adult minimal change NS patients. Zhang et al. [15] reported three children with INS received RTX alone as the initial treatment regimen, administered at 375 mg/m^2^ per week for 4 consecutive weeks. All three children achieved CR after 1–2 doses of RTX, with the longest duration of remission exceeding 24 months. Adverse reactions to RTX are rare, with occasional infusion-related reactions such as allergies, fever, and chest tightness. Some patients exhibited neutropenia and hypogammaglobulinaemia [10,12,15]. Consequently, we conducted a prospective, single-center, open-label, randomized controlled clinical trial aimed at evaluating whether the initial use of RTX monotherapy is superior to existing conventional treatment regimens for pediatric INS.

## 2. Methods

A single-center, randomized, parallel-controlled, open-label trial was conducted at The Children’s Hospital, Zhejiang University School of Medicine to explore the efficacy and safety evaluation of initial use of RTX in treating children with newly diagnosed NS. Eligible subjects were randomly assigned in a 1:1 ratio to the treated group (RTX monotherapy) or the control group (GC treatment). The study was conducted between 9 March 2023 and 10 April 2024. Clinical data was collected prospectively and reviewed according to schedule by the study team to ensure accuracy, completeness, and to perform data and safety monitoring.

The study protocol adheres to the Declaration of Helsinki and the clinical trial management regulations and laws of our country and has been approved by the Medical Ethics Committee of the Children’s Hospital affiliated with Zhejiang University School of Medicine (2022-IRB-229-A-04). This study was registered at clinical trials.gov (NCT05734794). All guardians of the study subjects provided valid informed written consent in keeping with the Helsinki accord.

### 2.1. Study design

This study employed a group randomization method, with every four participants forming a group and being randomly assigned in a 1:1 ratio to either the experimental group (RTX monotherapy) or the control group (standard treatment regimen). The randomization scheme was developed by an independent statistical team using SAS 9.4 software. Random seed numbers were generated and sorted to determine the intervention assignment for each participant. The independent statistical team placed the randomization scheme into sequentially numbered, opaque, sealed envelopes. The four grouping schemes for each condition were sequentially arranged in four opaque, sealed small envelopes numbered 1–4, which were then collectively placed into a larger envelope labeled with the corresponding group number. This envelope was managed by an independent center coordinator who did not participate in the implementation of the intervention or the outcome assessment.

The medical staff involved in the study assessed patients’ eligibility and enrolled them after obtaining informed consent. They subsequently contacted the center coordinator to obtain the allocation scheme for each patient. The center research coordinator at the center strictly adhered to the order in which the research subjects signed the informed consent form. They opened the envelopes in the numerical order indicated. They then informed the medical staff of the allocation scheme and completed a written registration form. Both the research subjects and the medical staff administering the research treatment were aware of the subjects’ group assignments. However, the medical staff responsible for outcome assessment and the data statistical analysis team remained completely unaware of the grouping.

### 2.2. Participants

Children aged 2–17 years with new-onset INS were included in the study, and their estimated glomerular filtration rate (eGFR) was ≥90 mL/min per 1.73 m^2^ at study entry. Exclusion criteria including: (1) Glomerular hematuria: urine red blood cell counts≥ 10/high power field (HP), ≥ 3 times within 2 weeks; (2) Continuous hypocomplementaemia (< 0.9 g/L); (3) Repeated or persistent hypertension (systolic and/or diastolic blood pressures measured greater than the 95^th^ percent of blood pressure in children matching sex, age and height ≥3 different time points); (4) Diagnosis of secondary NS, such as secondary to systemic lupus erythematosus, immunoglobulin A vasculitis (IgAV), diabetes mellitus, hepatitis B virus (HBV) infection; (5) Complicated with other kidney diseases, such as multiple renal cysts, ANCA vasculitis, urinary system abnormalities, etc; (6) With a family history of nephrotic syndrome, chronic glomerulonephritis, uremia, or other kidney diseases; (7) Other monogenic genetic diseases known as the effect the condition of nephrotic syndromes, such as Wilms’ tumor 1(WT1), NPHS2, LAMB2, PLCE1; (8) Congenital or acquired immunodeficiency, or patients with active tuberculosis, active Epstein-Barr virus and cytomegalovirus (CMV), acute hepatitis B, hepatitis C, HIV infection, deep fungal infection or other active infections; (9) Laboratory indicators were abnormal, such as moderate or severe neutropenia (≤1000/μL), moderate or severe anemia(hemoglobin < 9.0g/dL), Thrombocytopenia (platelet count < 100* 10^12^/L) or with abnormal hepatic function (alanin­e transaminase (ALT), aspartate aminotransferase (AST) or bilirubin >2.5*upper limit of normal value and continue to increase for 2 weeks); (10) Glucocorticoids or immunosuppressive medicine for other diseases within 3 months, such as use of cyclophosphamide, cyclosporine, tacrolimus, mycophenolate mofetil, *Tripterygium wilfordii*; (11) With tumor, severe cardiac failure, severe hepatologic diseases, hematological diseases, or other severe system diseases; (12) Patients who are known to be allergic to rituximab; (13) History of transplantation, excluding cornea or hair transplantation; (14) The attenuated live vaccine was inoculated within 1 month before enrollment; (15) Patients who participated in other clinical trials within three months before enrollment; (16) Patients are not suitable for inclusion in the trial by any investigator. All eligible patients were randomized into two groups according to a randomization chart: the RTX group and the GC group. In addition, general information was recorded, including age, sex, body mass index (BMI), course of disease and laboratory measurements.

### 2.3. Study intervention

The study lasted for 52 weeks. The treated group accepted 4 doses of RTX alone, while the control group was given the standard GC treatment regimen for NS. Subjects who achieve CR within 6 weeks of treatment in both groups will be followed up until the 52nd week after the study begins. We established 9 follow-up points at intervals of 0, 3, 6, 12, 18, 24, 36, and 52 weeks, as well as at the time of recurrence (within 3 days). Children who do not achieve CR after 6 weeks of treatment will require appropriate further clinical treatment plans.

#### 2.3.1. Treatment plan

##### 2.3.1.1. Treated group: RTX monotherapy

The regimen for RTX monotherapy consists of 375 mg/m^2^ body surface area (BSA) (maximum: 500 mg) administered once per week (within ±7 days) for a total of four doses. For the first 3 months of treatment, starting from the initiation of medication, trimethoprim-sulfamethoxazole (TMP/SMX) will be added at a dosage of 25 mg/kg/day, divided into two oral doses, taken for 3 days and then paused for 4 days. If the children exhibit any allergic reactions to TMP/SMX, the medication will be discontinued. During the treatment period, urine output, serum creatinine (SCr), serum albumin, 24-h urine protein quantification, urine protein/creatinine ratio (UPCR), and CD19 + B lymphocyte counts were monitored weekly during the first four infusions of RTX and at each subsequent visit, which will be conducted to promptly identify subjects achieving CR and the respective time points. This may lead to the following two scenarios:

Scenario 1: After completing 4 doses of RTX, the child does not achieve complete remission.

If the child exhibits no symptoms such as edema or oliguria and vital signs remain stable, CD19 + B lymphocyte counts will continue to be monitored without administering additional RTX, observing until the 6th week. Children achieving CR after 6 weeks of treatment will continue to be followed up until the 52nd week after the study begins.

Scenario 2: Complete remission occurs during the administration of the 4 doses of RTX.

The child will still need to complete all 4 doses of RTX treatment and will continue to receive TMP/SMX prophylactically for 3 months (using the same dosage as above), with follow-ups scheduled at specific time points until the 52nd week after the study begins.

##### 2.3.1.2. Control group: conventional steroid treatment regimen

The control group will receive a sufficient dose of prednisone at 2 mg/kg daily, not exceeding 60 mg, for 6 weeks, followed by a maintenance dose of 1.5 mg/kg every other day (not exceeding 50 mg every other day) for an additional 6 weeks. On the day of medication initiation, vitamin D and calcium supplements will be added (dosage can be adjusted based on serum calcium levels) for a total of 3 months. During the treatment period, regular monitoring of urine output, SCr, serum albumin, 24-h urine protein quantification, and urine protein/creatinine ratio will be conducted to promptly identify subjects achieving complete remission and the corresponding time points.

### 2.4. Outcomes

The primary outcome was the recurrence-free survival time from CR for participants who achieved CR within six weeks of treatment. CR was defined as UPCR <0.2 mg/mgCr and negative urine protein on three consecutive days. Relapse-free survival was defined as the duration from achieving CR to the diagnosis of relapse. Multiple secondary outcomes were set, including: (1) The CR rate of NS after 6 weeks of treatment; (2) The treatment failure rate after 6 weeks of treatment; (3) The duration from treatment initiation to CR of nephrotic syndrome; (4) The time taken for the CD19 + B lymphocyte count in the experimental group to first reach zero within 52 weeks of the study start; (5) The duration for the CD19 + B lymphocyte count in the experimental group to first recover to greater than 5 cells/μL after reaching exhaustion within 52 weeks of the study start; (6) The CD19 + B lymphocyte count at the time when the experimental group is definitively diagnosed with the first relapse; (7) The incidence of a CD19 + B lymphocyte count exceeding 5 cells/μL at the time of the first relapse diagnosis in the experimental group; (8) The duration from exhaustion to recovery above 5 cells/μL for the CD19 + B lymphocyte count in the experimental group leading up to the first relapse diagnosis (if the count exceeds 5 cells/μL, it will be remeasured monthly during follow-up); (9) The relapse rate of NS in children within 52 weeks of the study start; (10) The average number of relapses per child within 52 weeks of the study start; (11) The incidence of frequent relapses in children within 52 weeks of the study start; (12) The cumulative prednisone dosage (mg) administered to children within 52 weeks of the study start. Definitions and evaluation methods for related outcome indicators are detailed in Supplementary Material.

All indicators were determined regularly during the treatment period. The safety endpoint was the incidence of adverse events (AEs). All AEs were reported and coded in medical record.

### 2.5. Statistical analysis

Baseline characteristics were summarized by mean ± standard deviation (*SD*), median (interquartile range) and frequency (percentage) where appropriate. Statistical analysis was performed using SPSS version 27.0. For normally distributed data, results are presented as mean ± *SD*, with independent samples *t*-test being utilized for intergroup comparisons. Non-normally distributed data, on the other hand, are usually presented as median, with the rank-sum test being used for intergroup comparisons. All statistical tests were two-sided, with a significance level set at *α* = 0.05; *p* < 0.05 indicates statistical significance. GraphPad Prism 10.0 software was employed to analyze the cumulative prednisone dosage.

## 3. Results

### 3.1. Enrollment and follow-up

**Figure 1.**
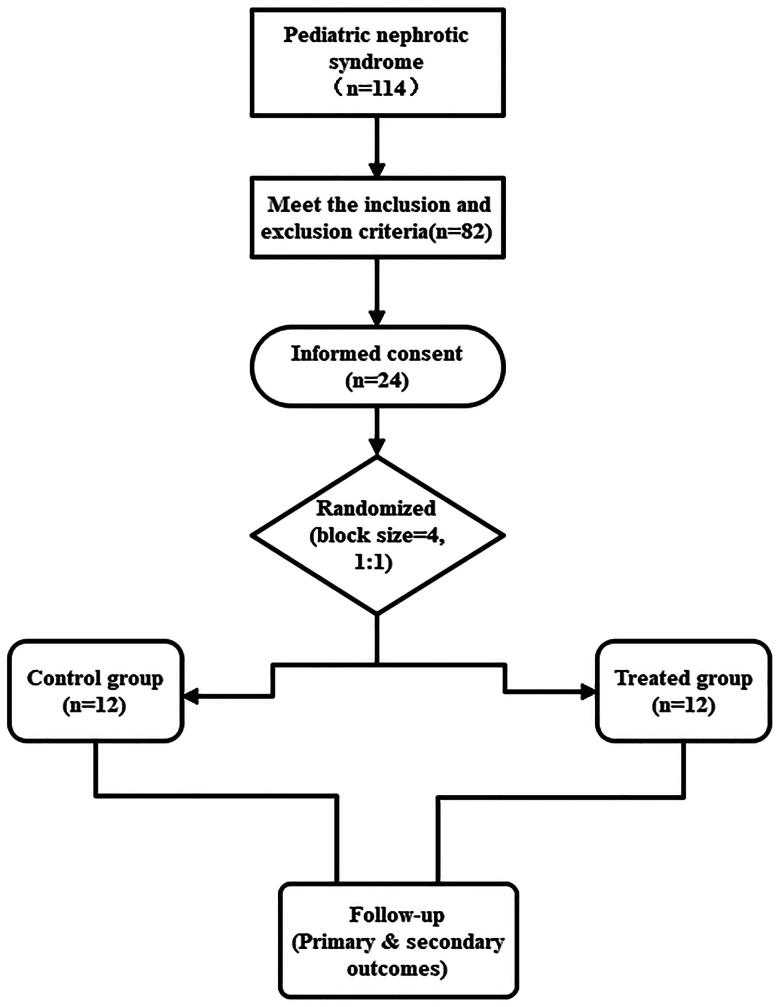
Flow diagram of enrollment, randomization, and follow-up of patients.

Between March 2023 and April 2024, a total of 114 patients with newly diagnosed NS were admitted to our center, and 82 patients met the inclusion and exclusion criteria. Treatment options were discussed with both patients and their families. 24 patients consented to participate in the study and were randomized to the RTX group (*N* = 12) and the GC group (*N* = 12) (Figure 1). All enrolled patients were regularly followed up, with no withdrawals or losses to follow-up reported. The follow-up period extended until 31 December 2024, with 15 patients monitored for over 52 weeks. The follow-up duration in the GC group ranged from 38 to 94 (median, 61.0) weeks and 39 to 84 (median, 61.5) weeks in the RTX group, with no statistically significant difference between the two groups (*p* > 0.05). All children exhibited normal renal function throughout the follow-up period and did not experience thrombosis or acute kidney injury (AKI). The patient did not receive angiotensin converting enzyme inhibitors (ACEI) or angiotensin receptor blockers (ARB) medications during the treatment period. Additionally, no immunosuppressants were administered alongside the study drug, and there was no use of any Chinese herbal medicine.

The basic characteristics of the two groups are presented in Table 1. Both groups were predominantly male, but no statistically significant difference in gender was observed. The median age of children in the control group was 59.5 months old, with no statistical difference noted between the groups. The average BMI of children in the RTX group was significantly lower than that of the GC group. Furthermore, compared to the RTX group, the GC group demonstrated higher levels of UPCR and more pronounced hypercholesterolemia, with a statistically significant difference between the two groups. No statistically significant differences were found regarding CD19 + B cell counts, urinary protein quantification, serum albumin levels, triglycerides, D-Dimer levels at onset, and the time to initiate treatment.

**Table 1.** Baseline characteristics of the subjects.

	All (*n* = 24)	Treated (*n* = 12)	Control (*n* = 12)	*p*
Male (*n*)	19	11	8	*0.132*
Median age (months)	59 (24–157)	47.5 (34–98)	59.5 (24–157)	*0.443*
BMI (kg/m^2^)	16.51 ± 1.86	15.69 ± 1.23	17.33 ± 2.06	0.027*
IgG (g/L)	3.51 ± 2.06	3.33 ± 1.61	3.69 ± 2.48	*0.681*
IgM (g/L)	1.38 ± 0.76	1.43 ± 0.68	1.33 ± 0.86	*0.743*
IgA (g/L)	1.37 ± 0.49	1.30 ± 0.58	1.44 ± 0.39	*0.496*
CD19 + B cell counts (/uL, median)	601.9	630	443	*0.478*
24-urinary protein (mg/day)	5,097 ± 3127	4,204 ± 3300	5,590 ± 2795	*0.167*
24-urinary protein (mg/kg per d, median)	198.25	174.17	209.65	*0.443*
UPCR (mg/mgCr, median)	10.32	6.64	13.63	0.045*
Serum albumin (g/L)	17.53 ± 3.34	17.67 ± 3.08	17.40 ± 3.72	*0.845*
Cholesterol (mmol/L, median)	10.78	9.6	12.62	0.001*
Triglyceride (mmol/L)	3.24 ± 1.79	3.14 ± 1.42	3.35 ± 2.16	*0.779*
D dimer (mg/L, median)	0.95	0.83	1.01	*0.41*
Course of disease (d, median)	8	8.5	8	*0.799*

BMI: body mass index; UPCR: urinary protein/creatinine ratio.

### 3.2. Primary outcomes

All 12 patients in the GC group achieved CR within 6 weeks, but 9 patients relapsed during follow-up. The longest recurrence-free survival time was 94 weeks (median 33.5 weeks) in the GC group. Patients with CR treated with RTX did not relapse during follow-up, and the longest relapse-free survival was 79 weeks (median 63 weeks).

### 3.3. Secondary outcomes

**Figure 2.**
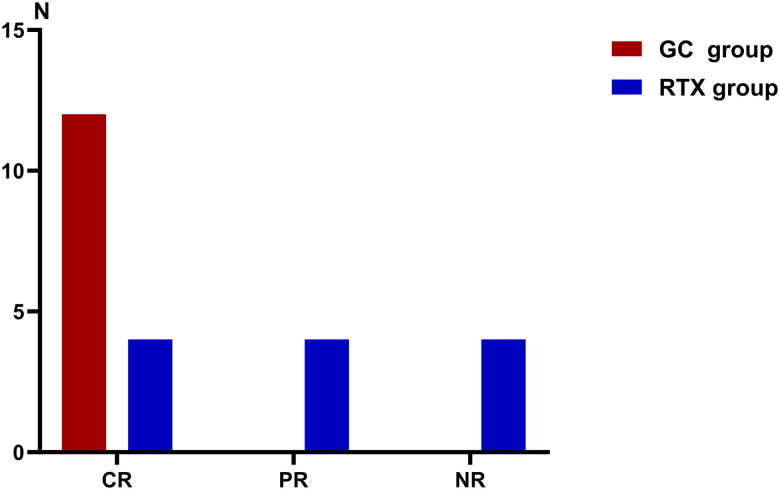
The status of disease response within 6 weeks of treatment.

4(1/3) patients in the RTX group achieved CR, 4(1/3) patients exhibited partial remission (PR), and four (1/3) were deemed non-remission (NR) within 6 weeks of treatment (see Figure 2). Two of the four (2/4) patients who were classified as NR received only 3 doses of RTX, and prednisone (2 mg/kg/day) was administered subsequently, leading to CR. Four children with PR received 4 doses of RTX and continued to be monitored. However, after 2 weeks of observation, they developed significant proteinuria or edema, prompting the decision to initiate treatment with a prednisone, which ultimately led to negative urinary protein. All 12 (100%) children in the GC group achieved CR. The rate of CR was significantly different between the two groups (*p* > 0.05).

In the RTX group, the time from initial treatment to CR was 6, 14, 17, and 20 days, respectively, resulting in an average remission time of 14.25 days. In contrast, the remission time for the 12 control children ranged from 6 to 16 days, with an average of 9.5 days. All children achieved B cell depletion after 4 infusions of RTX, and 8 of them achieved B cell depletion after 2 infusions. The first exhaustion of CD19+ B lymphocyte counts in the RTX group occurred between 6 and 41 days, with an average time of 18.75 days (counted from first infusion). The average recovery time for CD19+ B lymphocytes was 22 weeks, with a range of 18–36 weeks. Patients receiving RTX alone have not experienced a relapse. Therefore, we did not observe CD19+ B lymphocyte counts during relapse in the RTX group.

The follow-up durations for the 4 patients in the RTX group with CR were 79 weeks, 78 weeks, 48 weeks, and 43 weeks, respectively. During the follow-up period, none of these patients experienced a recurrence. The remaining 8 patients who accepted prednisone gradually reduced their prednisone dosage. Among them, two (2/12) cases experienced relapses at the 28th and 61st weeks following the initiation of treatment, respectively. In the control group, 9 (9/12) patients relapsed during the follow-up, with the first relapses occurring in the 11th, 16th, 21st, 23rd, 28th, 28th, 33rd, 39th, and 45th weeks after treatment initiation. 4 patients (4/12) were categorized as frequently relapsing nephrotic syndrome (FRNS). One patient relapsed twice without discontinuation of steroids, and one patient had two recurrences, both due to the self-withdrawal of steroids. Of the 9 patients with recurrence, 5 had recurrence following an upper respiratory tract infection, while the other 4 had recurrence without an obvious trigger.

**Figure 3.**
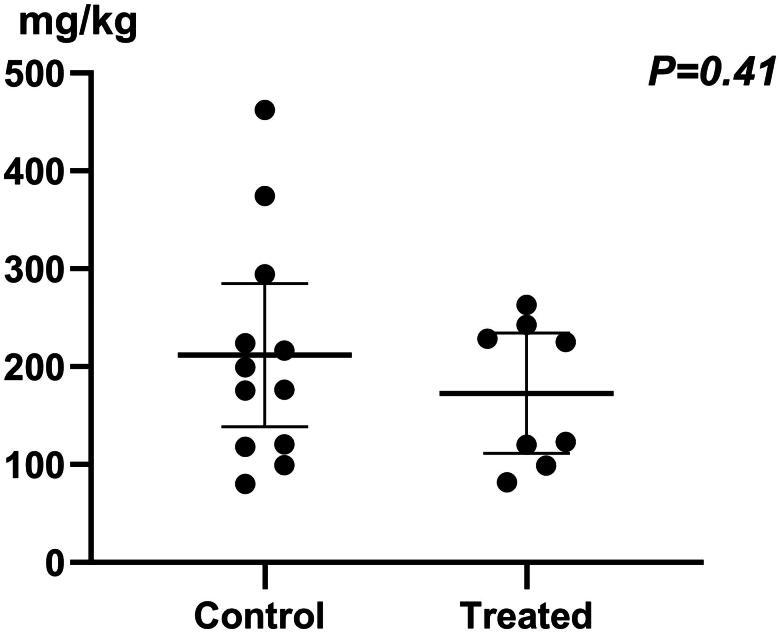
The cumulative prednisone dosage (mg/kg) administered to children following the start of the study.

The cumulative steroid dosage was 211.80 mg/kg (80.41–462.39) in the GC group, compared to 172.86 mg/kg (81.87–263.13) in the RTX group. The difference was not statistically significant (*p* = 0.41) (Figure 3).

### 3.4. Safety outcomes

Neither group experienced any serious AEs, and renal function remained stable throughout the follow-up period. In the RTX group, 9 patients developed rashes, coughs, and throat discomfort during infusion. Five patients experienced fever during the infusion, with 2 showing symptoms of an upper respiratory tract infection, including cough and nasal congestion. No serious infectious events were recorded. In the GC group, one patient had ocular hypertension during treatment, and 3 children experienced hairness. We regularly monitored the serum IgG levels and neutrophil counts of both groups. Definitions of the relevant grades are provided in Supplementary Material. Baseline hypogammaglobulinemia was observed in 10 and 11 patients in the GC and RTX groups, respectively. In patients receiving RTX, IgG levels decreased from baseline in 6 individuals, with reductions ranging from 7.8% to 79.1%. However, persistent hypogammaglobulinemia was not observed during follow-up. During the follow-up period, no patients in the GC group developed neutropenia. In the RTX group, there was one case each of mild, moderate, and severe neutropenia, with no statistically significant differences between the groups.

## 4. Discussion

This randomized controlled trial (RCT) examined the effectiveness and safety of initial RTX treatment in children newly diagnosed with NS. Our results show that only 33.3% of patients achieved CR following the initial RTX treatment. Additionally, 75% of the children experienced mild allergic reactions during the treatment process. Our data indicated that 87.5% (21/24) of the patients exhibited low baseline IgG levels, which was slightly higher than what has been reported in the literature [16]. This observation may be attributed to IgG loss through the glomerular filtration barrier during the acute phase of the disease. Following RTX infusion, 50% of individuals experienced aggravated hypogammaglobulinemia. During the subsequent follow-up period, we observed a gradual recovery of IgG levels. However, no severe infection events were reported. It is important to note that the four children who achieved CR did not experience relapse during the follow-up period. This highlights the effectiveness of RTX in preventing disease recurrence. Individuals with PR or NR demonstrated steroid sensitivity when GCs were added, and no relapses occurred during the subsequent tapering of GCs.

Increasing evidence suggests that podocyte autoantibodies play a significant role in childhood INS and are associated with disease activity [7,17]. Children with INS primarily driven by B cell immunity may respond favorably to RTX. It is crucial to screen for podocyte autoantibodies prior to initiating treatment. RTX has been used for more than ten years to prevent relapses in FRNS or steroid-dependent nephrotic syndrome (SDNS) [1]. A number of retrospective reports have indicated that 30–80% of children experienced either complete or partial remission following the use of RTX [18–20]. Compared with traditional treatments, studies have confirmed that RTX administration allowed for steroid withdrawal, prevented recurrences, and promoted growth [21–23]. Recently, a large, multi-ethnic pediatric cohort demonstrated that RTX enhances remission in a subset of children with steroid-resistant nephrotic syndrome (SRNS). However, most patients received concomitant therapies such as steroids and non-steroidal immunosuppressants. Meanwhile, there are limited studies on the use of RTX alone for the initial treatment of primary NS, most of which are confined to case reports [13–14].

RTX may modulate the interaction between B cells and T cells [24]. The pathogenesis of NS is highly complex, often involving the interplay of T cells, B cells, and cytokines, along with individual variations [2]. INS may not solely be driven by B cells or antibodies, which accounts for the low rate of CR achieved with RTX monotherapy in our study. Furthermore, RTX has a molecular weight of approximately 145 kDa. It remains unclear whether RTX can permeate the glomerular filtration membrane when podocytes are damaged. Additionally, we did not measure the blood concentration of RTX. It is worth noting that 5 patients in the experimental group had infection during the infusion, which was not conducive to the remission of the condition.

Our study found that the average time for B cell depletion after administering RTX was 18.75 days, with B cell recovery occurring around 22 weeks, consistent with previous studies [1]. Due to the low rate of CR observed in individuals who received initial RTX, we do not recommend RTX as a first-line monotherapy for INS. However, for specific populations with contraindications to GC administration, such as those with poorly controlled diabetes, obesity (BMI > 30 kg/m^2^), peptic ulcers, serious osteoporosis, uncontrolled tuberculosis, viral hepatitis, and psychiatric disorder, RTX may be considered as an initial treatment option [25]. Our findings indicated a lower cumulative GC dose in the RTX group compared to the control group, although the difference was not statistically significant. All participants in the control group were defined as SSNS, and those in the RTX group who did not achieve CR also showed signs of steroid-sensitivity after steroid administration. In the GC group, 9 cases (66.7%) experienced recurrence during the follow-up period, with 4 cases being classified as FRNS. Conversely, our follow-up data showed that children who received sequential steroid treatment after RTX acquired a longer relapse-free survival time compared to those treated solely with steroids. Although they were subjected to prolonged steroid therapy, previous studies have indicated that longer courses of treatment have no advantage over shorter ones in terms of preventing relapse [3,26]. The duration of steroid therapy remains a subject of debate. The sequential use of GCs and RTX may yield better outcomes, mitigating steroid side effects and lowering the recurrence rate.

There are still several unanswered questions regarding RTX, such as the best timing for its administration, specific treatment plans, and the lack of clear guidelines for reducing GCs after RTX. Previous research has indicated that the chimeric nature of RTX may play a role in the development of anti-RTX antibodies, potentially reducing the circulating levels of RTX and resulting in a shorter duration or lack of depletion of B cells [27]. Consequently, the presence of anti-RTX antibodies may hinder the efficacy of subsequent infusions and increase the risk of infusion-related reactions [28]. Although the effectiveness of RTX highlights the involvement of B cells in the pathophysiology of the disease, consensus is lacking regarding the relationship between B cells clearance and disease remission, as well as between B cells reconstitution and disease relapse.

This study is a prospective, randomized, controlled trial. Our findings confirm that the CR rate in individuals initially treated with RTX is not significant, further supporting the use of GCs as the primary treatment for children with INS. Importantly, no serious adverse events were reported following RTX administration, consistent with previous studies that affirm the safety of RTX. However, this study is limited by its single-center design, small sample size, and short follow-up duration. The trial was terminated prematurely for ethical reasons, as the anticipated clinical effects were not achieved and not all participants completed the minimum follow-up period of 52 weeks. Our study did not investigate the mechanism of action of RTX, particularly its effects on T and B cell interactions. Nonetheless, our findings reflect the real-world clinical practice in the initial treatment of pediatric INS, and provide a reference to pediatric nephrologists. We anticipate future research aimed at developing individualized treatments based on the immunological characteristics of patients to enhance treatment efficacy. Therefore, further multicenter, large-scale studies are necessary to evaluate the initial use of RTX in the treatment of children with INS.

## Conclusion

RTX alone has not demonstrated clinical benefits in children with INS. However, it was not associated with serious adverse effects, and the overall recurrence rate was lower when RTX was administered.

## Supplementary Material

Revised_Supplementary_Material.docx

## Data Availability

The datasets used and analyzed during the current study are available from the corresponding author upon reasonable request.

## References

[1] Trautmann A, Boyer O, Hodson E, et al. IPNA clinical practice recommendations for the diagnosis and management of children with steroid-sensitive nephrotic syndrome. Pediatr Nephrol. 2023;38(3):877–919. doi:10.1007/s00467-022-05739-3. 36269406 PMC9589698

[2] Vivarelli M, Gibson K, Sinha A, et al. Childhood nephrotic syndrome. Lancet. 2023;402(10404):809–824. doi:10.1016/S0140-6736(23)01051-6. 37659779

[3] Veltkamp F, Rensma LR, Bouts A. Incidence and relapse of idiopathic nephrotic syndrome: meta-analysis. Pediatrics. 2021;148(1):e2020029249. doi:10.1542/peds.2020-029249. 34193618

[4] Grennan D, Wang S. Steroid side effects. JAMA. 2019;322(3):282. doi:10.1001/jama.2019.8506. 31310300

[5] Noone DG, Iijima K, Parekh R. Idiopathic nephrotic syndrome in children. Lancet. 2018;392(10141):61–74. doi:10.1016/S0140-6736(18)30536-1. 29910038

[6] Horinouchi T, Nozu K, Iijima K. An updated view of the pathogenesis of steroid-sensitive nephrotic syndrome. Pediatr Nephrol. 2022;37(9):1957–1965. doi:10.1007/s00467-021-05401-4. 35006356 PMC9307535

[7] Hengel FE, Dehde S, Lassé M, et al. Autoantibodies targeting nephrin in podocytopathies. N Engl J Med. 2024;391(5):422–433. doi:10.1056/NEJMoa2314471. 38804512

[8] Kallash M, Smoyer WE, Mahan JD. Rituximab use in the management of childhood nephrotic syndrome. Front Pediatr. 2019;7:178. doi:10.3389/fped.2019.00178. 31134169 PMC6524616

[9] Iijima K, Sako M, Nozu K. Rituximab for nephrotic syndrome in children. Clin Exp Nephrol. 2017;21(2):193–202. doi:10.1007/s10157-016-1313-5. 27422620 PMC5388729

[10] Sinha A, Bagga A. Rituximab therapy in nephrotic syndrome: implications for patients’ management. Nat Rev Nephrol. 2013;9(3):154–169. doi:10.1038/nrneph.2012.289. 23338210

[11] Chan EY, Yu E, Angeletti A, et al. Long-term efficacy and safety of repeated rituximab to maintain remission in idiopathic childhood nephrotic syndrome: an international study. J Am Soc Nephrol. 2022;33(6):1193–1207. doi:10.1681/ASN.2021111472. 35354600 PMC9161790

[12] Ravani P, Ponticelli A, Siciliano C, et al. Rituximab is a safe and effective long-term treatment for children with steroid and calcineurin inhibitor-dependent idiopathic nephrotic syndrome. Kidney Int. 2013;84(5):1025–1033. doi:10.1038/ki.2013.211. 23739238 PMC3816123

[13] Medjeral-Thomas NR, Lawrence C, Condon M, et al. Randomized, controlled trial of tacrolimus and prednisolone monotherapy for adults with de novo minimal change disease: a multicenter, randomized, controlled trial. Clin J Am Soc Nephrol. 2020;15(2):209–218. doi:10.2215/CJN.06180519. 31953303 PMC7015084

[14] Xu R, Hu H, Xu H, et al. Initial rituximab monotherapy for adult indiopathic nephrotic syndrome with minimal change lesion pattern. Nephrol Dial Transplant. 2024;39(5):893–895. doi:10.1093/ndt/gfae012. 38218590

[15] Zhang X, Jin Y, Li Q, et al. Successful treatment of new-onset pediatric nephrotic syndrome with rituximab as a first-line therapy. Kidney Int Rep. 2022;7(12):2750–2751. doi:10.1016/j.ekir.2022.10.016. 36506238 PMC9727513

[16] Chan EY, Ma AL, Tullus K. Hypogammaglobulinaemia following rituximab therapy in childhood nephrotic syndrome. Pediatr Nephrol. 2022;37(5):927–931. doi:10.1007/s00467-021-05345-9. 34999985

[17] Ye Q, Zhou C, Wang D, et al. Seven novel podocyte autoantibodies were identified to diagnosis a new disease subgroup-autoimmune Podocytopathies. Clin Immunol. 2021;232:108869. doi:10.1016/j.clim.2021.108869. 34600127

[18] Sinha A, Bhatia D, Gulati A, et al. Efficacy and safety of rituximab in children with difficult-to-treat nephrotic syndrome. Nephrol Dial Transplant. 2015;30(1):96–106. doi:10.1093/ndt/gfu267. 25121488

[19] Basu B, Mahapatra T, Mondal N. Mycophenolate mofetil fol-lowing rituximab in children with steroid-resistant nephroticsyndrome. Pediatrics. 2015;136(1):e132–e139. doi:10.1542/peds.2015-0486. 26101364

[20] Kamei K, Okada M, Sato M, et al. Rituximab treatment combined with methylprednisolone pulse therapy and immunosuppressants for childhood steroid-resistantnephroticsyndrome. Pediatr Nephrol. 2014;29(7):1181–1187. doi:10.1007/s00467-014-2765-z. 24500706

[21] Iijima K, Sako M, Nozu K, et al. Rituximab for childhood-onset, complicated, frequently relapsing nephrotic syndrome or steroid-dependent nephrotic syndrome: a multicentre, double-blind, randomised, placebo-controlled trial. Lancet. 2014;384(9950):1273–1281. doi:10.1016/S0140-6736(14)60541-9. 24965823

[22] Ravani P, Rossi R, Bonanni A, et al. Rituximab in children with steroid-dependent nephrotic syndrome: a multicenter, open-label, noninferiority, randomized controlled trial. J Am Soc Nephrol. 2015;26(9):2259–2266. doi:10.1681/ASN.2014080799. 25592855 PMC4552120

[23] Ruggenenti P, Ruggiero B, Cravedi P, et al. Rituximab in steroid- dependent or frequently relapsing idiopathic nephrotic syn-drome. J Am Soc Nephrol. 2014;25(4):850–863. 24480824 10.1681/ASN.2013030251 PMC3968490

[24] Chan EY, Sinha A, Yu ELM, et al. An international, multi-center study evaluated rituximab therapy in childhood steroid-resistant nephrotic syndrome. Kidney Int. 2024;106(6):1146–1157. doi:10.1016/j.kint.2024.09.011. 39395629

[25] Kidney Disease: Improving Global Outcomes (KDIGO) Glomerular Diseases Work Group. KDIGO 2021 Clinical Practice Guideline for the Management of Glomerular Diseases. Kidney Int. 2021;100(4S):S1–S276. doi:10.1016/j.kint.2021.05.021. 34556256

[26] Hodson EM, Hahn D, Craig JC. Corticosteroids for the initial episode of steroid-sensitive nephrotic syndrome. Pediatr Nephrol. 2015;30(7):1043–1046. doi:10.1007/s00467-015-3106-6. 25912994

[27] Bertrand Q, Mignot S, Kwon T, et al. Anti-rituximab antibodies in pediatric steroid-dependent nephrotic syndrome. Pediatr Nephrol. 2022;37(2):357–365. doi:10.1007/s00467-021-05069-w. 34132894

[28] Zurowska A, Drozynska-Duklas M, Topaloglu R, et al. Rituximab-associated hypogammaglobulinemia in children with idiopathic nephrotic syndrome: results of an ESPN survey. Pediatr Nephrol. 2023;38(9):3035–3042. doi:10.1007/s00467-023-05913-1. 37014530 PMC10432325

