# Supplementary material for: The efficacy and safety of rituximab monotherapy in the new onset pediatric idiopathic nephrotic syndrome: a randomized controlled clinical trial
Source: Ren Fail. 2025 May 6;47(1):2499902. doi: 10.1080/0886022X.2025.2499902 (PMC12057790; doi:10.1080/0886022X.2025.2499902)
Supplement: Revised_Supplementary_Material.docx [file IRNF_A_2499902_SM2698.docx]

Interventions


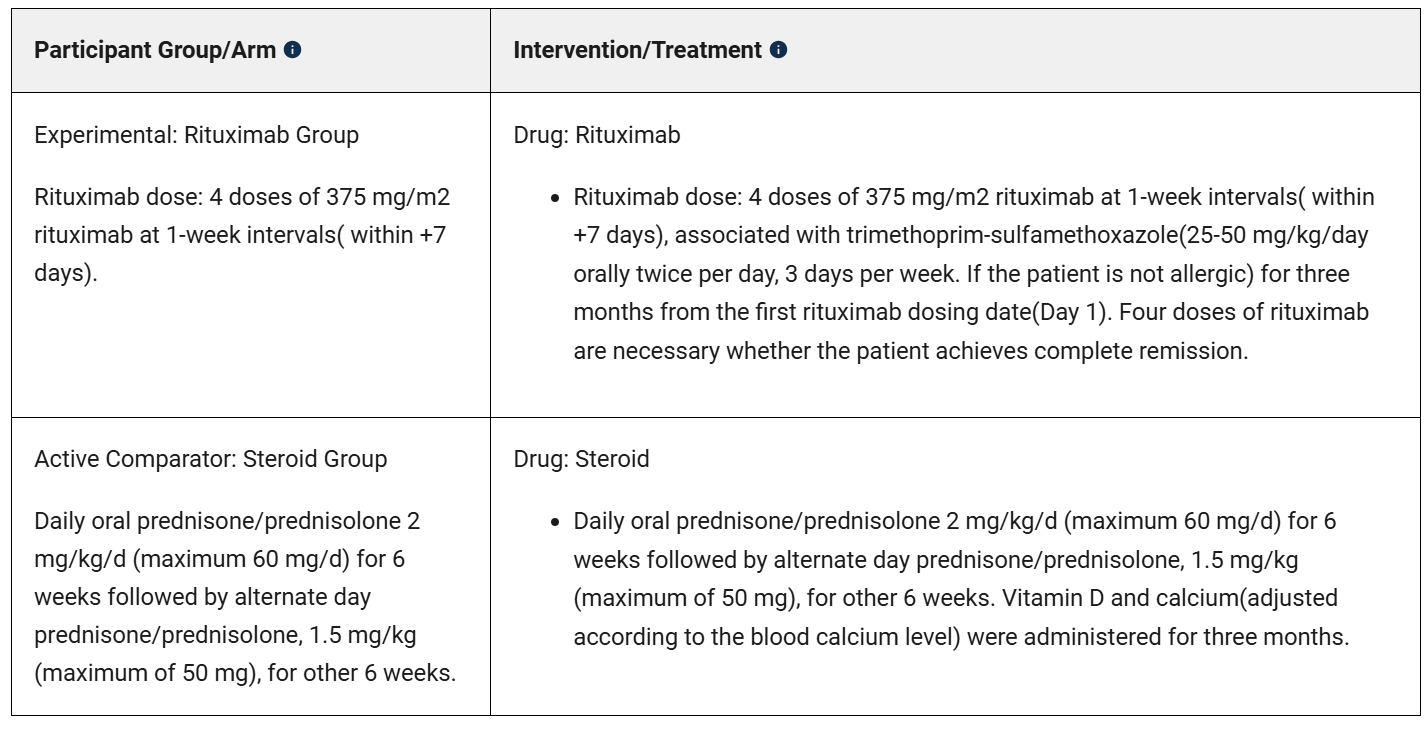


Primary outcome Measure


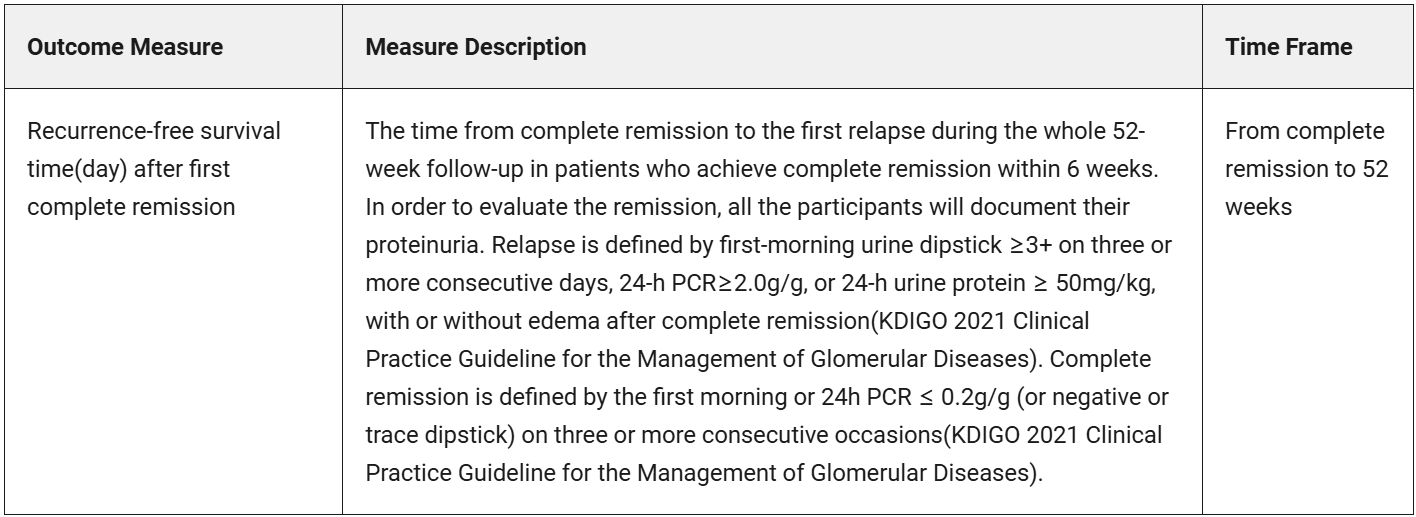


Secondary outcome Measure


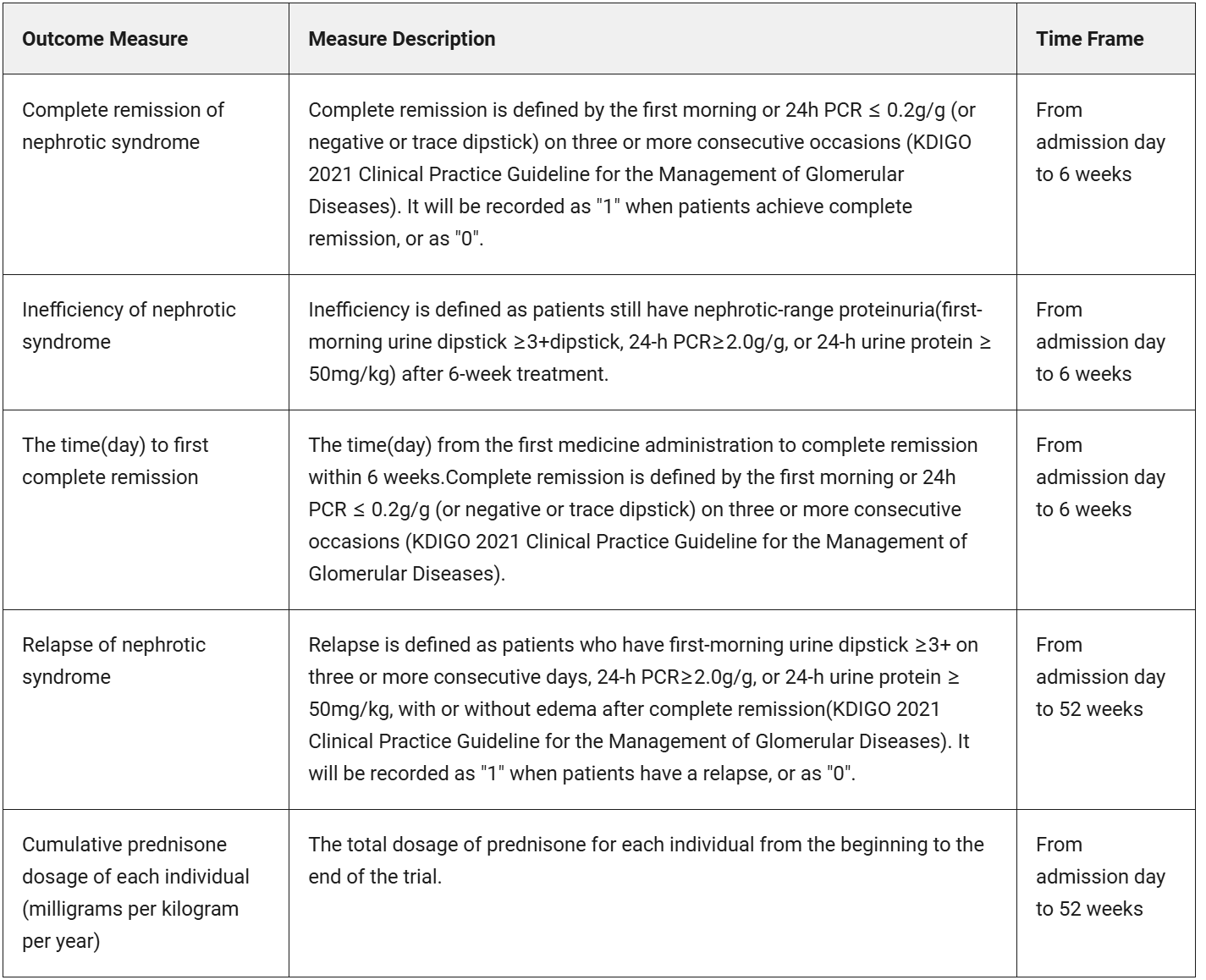


Definitions and Assessment Methods for Outcome Indicators:
**(1) Degree of Remission :**

**Complete Remission:** Continuous urinary protein/creatinine ratio <0.2 mg/mgCr for a minimum of 3 days, or morning urine analysis indicating negative urinary protein;

**Partial Remission:** Urinary protein/creatinine ratio between 0.2 mg/mgCr and 2 mg/mgCr; morning urine analysis showing urinary protein < ++, absence of edema, and serum albumin > 30 g/L.

**Ineffective:** Persistent nephrotic-level proteinuria after 6 weeks of treatment, defined as 24-hour urinary protein ≥ 50 mg/kg, urinary protein/creatinine > 2 mg/mgCr, or morning urine analysis indicating urinary protein ≥ +++.
**(2) The time(day) to first complete remission:**The time(day)from the first medicine administration to complete remissionwithin 6 weeks.

1. **Relapse:** Reappearance of nephrotic-level proteinuria for 3 consecutive days, defined as morning urinary protein ≥ +++, or 24-hour urinary protein ≥ 50 mg/kg or urinary protein/creatinine (mg/mg) ≥ 2.0, with or without edema. (Data type: Binary outcome)
   **(4) Frequent Relapse:** Occurrence of relapse ≥ 2 times within 6 months of initial diagnosis or relapse ≥ 4 times within any year. (Data type: Binary outcome)

**(5) Cumulative prednisonedosage of each individual(milligrams per kilogramper year):** The total dosage of prednisone for each individual from the beginning to the end of the trial.
**(6) Hypogammaglobulinemia Grading:**

Mild hypogammaglobulinemia (400–599 mg/dL);

Moderate hypogammaglobulinemia (200–399 mg/dL);

Severe hypogammaglobulinemia (0–199 mg/dL).

1. **Neutropenia Grading:**

Neutropenia (1000–1500/μL);

Moderate neutropenia (500–1000/μL);

Severe neutropenia (<500/μL).
